# Supplementary material for: The Hippo–Yki Signaling Pathway Positively Regulates Immune Response against Vibrio Infection in Shrimp
Source: Int J Mol Sci. 2022 Oct 7;23(19):11897. doi: 10.3390/ijms231911897 (PMC9569791; doi:10.3390/ijms231911897)
Supplement: Supplementary file 1 [file ijms-23-11897-s001.zip › Supplementary Table S1.pdf]

Table S1 Summary of primers used in this study

Primers	Sequences(5' to 3')
**For cDNA cloning**	
Hippo-ORF-F	ATGACGAGTCTGGAGAACAAGGAGA
Hippo-ORF-R	GAAATTCTGCTGTCTCTTCCTTTTCTGG
Wts-ORF-F	ATGGCCCCTCAGATGCCTCAT
Wts-ORF-R	TACATAGACTGGATGTTGACTGTCCTTC
YAP-ORF-F	ATGGCCAGCTCGAACAAGGATG
YAP-ORF-R	CAGCCACGTATGGAGATTATCTATTTTGG
**RT-PCR analysis**	
Hippo-F	GCCCCCTTACGGTGACATT
Hippo-R	GAACTCGGGTTGCCACTGAT
Wts-F	TGGCTCACATACCAATAACTCAA
Wts-R	GGGATGACTCAGACCTGGTGTTA
YAP-F	ACTACCACAGACATCCACACGC
YAP-R	GGGAGTATGAGGGAGGGAGTAA
Dorsal-F	TTGCGACCACCAGACAAGAG
Dorsal-R	GCAAGGTAACGACTAATCTTCTCTG
STAT-F	CTTCGCCATCCGTCCTCTAG
STAT-R	GGCTTGATCCTTAGGCACATTC
Relish-F	CTGCTTCTCCATACTCAGACCAC
Relish-R	CTGTGGCTGCTCCAGTATTTG
EF-1a-F	CCTATGTGCGTGGAGACCTTC
EF-1a-R	GCCAGATTGATCCTTCTTGTTGAC
Vpa-16s	GGTGTAGCGGTGAAATGCGTAG
Vpa-16s	CCACAACCTCCAAGTAGACATCG
**Promoter cloning**	
pGL3-Hippo-F	GACATAAGTGAACAAGAGGGAAAAGACAG
pGL3-Hippo-R	CTTGCAGGTTCTTCACACGGGA
pGL3-Wts-F	GATAGATACATGGTTCTGAAATACATCGC
pGL3-Wts-R	TACCTCCTTTTCCTCTCGACCTCT
pGL3-YAP-F	GATAATGAACTATGGCAATCGCAATCTG
pGL3-YAP-R	CTTGGCTACTTAGGGTCAACACTCA
**dsRNA production**	
dsHippo-F:	GGCAGAACCAAATCAAGCATA
dsHippo-R :	TCTTCTAAAGGCAACTGGGATG
dsHippo-T7-F:	GGATCCTAATACGACTCACTATAGG GGCAGAACCAAATCAAGCATA
dsHippo-T7-R:	GGATCCTAATACGACTCACTATAGG TCTTCTAAAGGCAACTGGGATG
dsWts-F:	AGTGAAGTAGTGCCAACCACAGAT
dsWts-R :	GAGATGATGTGGCAGTAGAAAGG
dsWts-T7-F:	GGATCCTAATACGACTCACTATAGG AGTGAAGTAGTGCCAACCACAGAT
dsWts-T7-R:	GGATCCTAATACGACTCACTATAGG GAGATGATGTGGCAGTAGAAAGG
dsYAP-F:	ACGCAAGTGAGCCACCAGT
dsYAP-R :	TCCGTGGACAGGTCATCATT
dsYAP-T7-F:	GGATCCTAATACGACTCACTATAGG ACGCAAGTGAGCCACCAGT
dsYAP-T7-R:	GGATCCTAATACGACTCACTATAGG TCCGTGGACAGGTCATCATT
dsSTAT-F	TCAGTATGCCCAGTCCTT
dsSTAT-R	CCTAACTCTTTCCGTCTCC
T7-dsSTAT-F	GGATCCTAATACGACTCACTATAGGTCAGTATGCCCAGTCCTT
T7-dsSTAT-R	GGATCCTAATACGACTCACTATAGGCCTAACTCTTTCCGTCTCC

dsDorsal-F:	TGTATCTCTTCGGAGGTTGGAC
dsDorsal-R :	AACATTGTGCTGGGCTGACT
dsDorsal-T7-F:	GGATCCTAATACGACTCACTATAGGTGTATCTCTTCGGAGGTTGGAC
dsDorsal-T7-R:	GGATCCTAATACGACTCACTATAGGAACATTGTGCTGGGCTGACT
dsRelish-F	AGAGGTGACAGAGGTGGGAT
dsRelish-R	CTTGCATGGGTTATCAACTC
T7-dsRelish-F	GGATCCTAATACGACTCACTATAGGAGAGGTGACAGAGGTGGGAT
T7-dsRelish-R	GGATCCTAATACGACTCACTATAGGCTTGCATGGGTTATCAACTC
GFP-F	ATGGTGAGCAAGGGCGAGGAG
GFP-R	TTACTTGTACAGCTCGTCCATGCC
T7-GFP-F	GGATCCTAATACGACTCACTATAGGATGGTGAGCAAGGGCGAGGAG
T7-GFP-R	GGATCCTAATACGACTCACTATAGGTTACTTGTACAGCTCGTCCATGCC
