# Supplementary figures and images for: The Hippo–Yki Signaling Pathway Positively Regulates Immune Response against Vibrio Infection in Shrimp
Source: Int J Mol Sci. 2022 Oct 7;23(19):11897. doi: 10.3390/ijms231911897 (PMC9569791; doi:10.3390/ijms231911897)

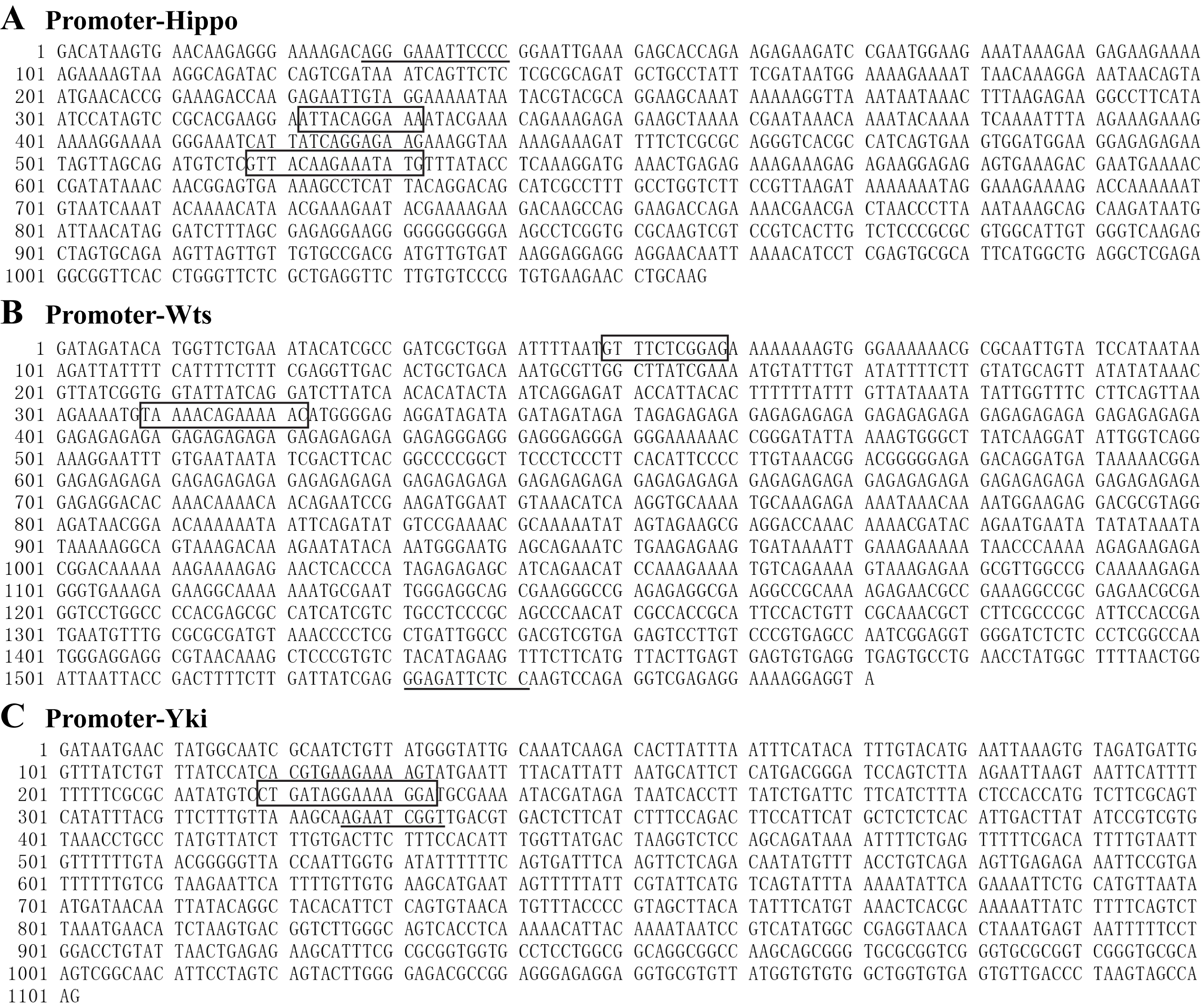

Supplement: Supplementary file 1 [file ijms-23-11897-s001.zip › Supplementary Figure S1.tif]

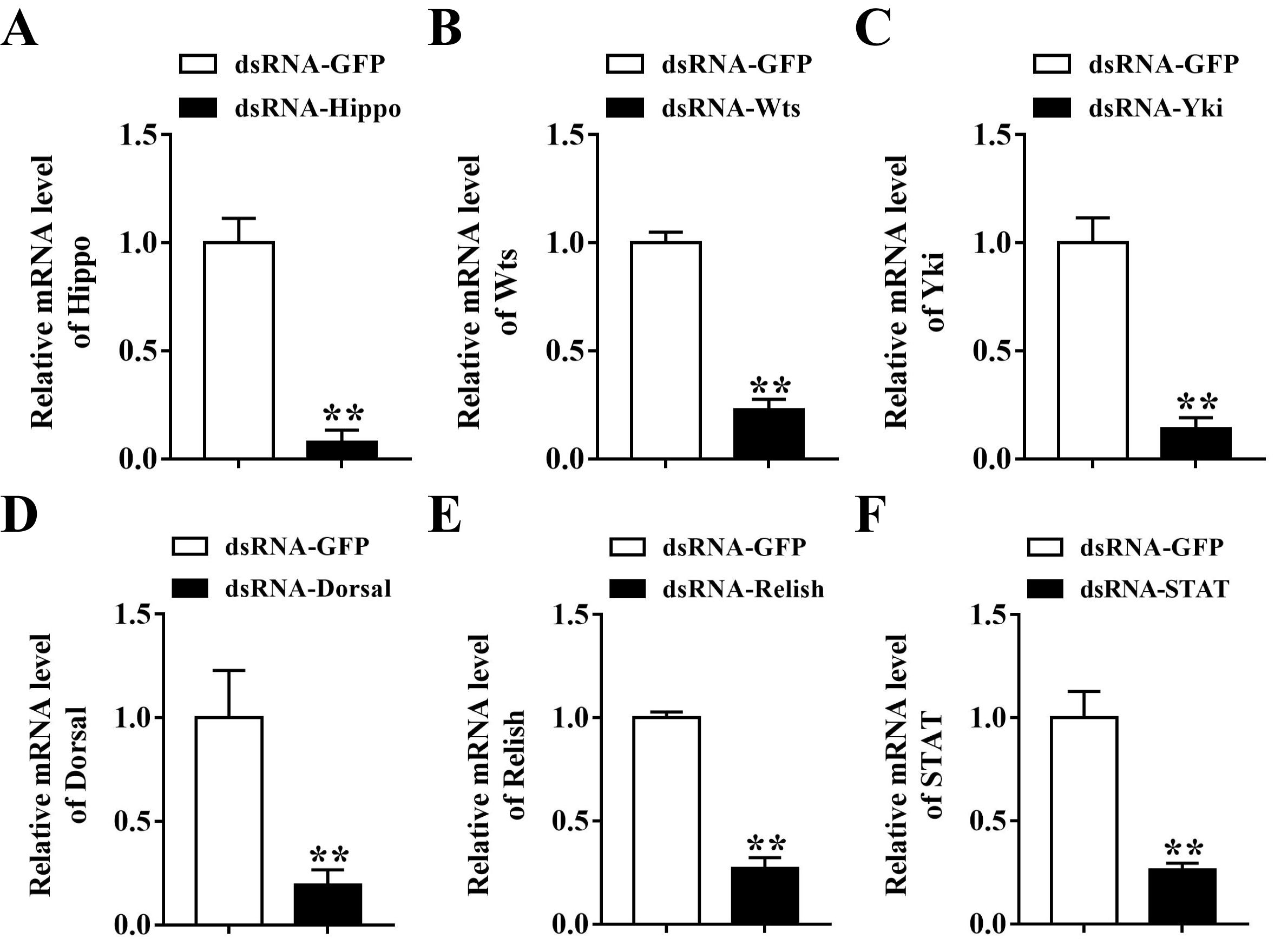

Supplement: Supplementary file 1 [file ijms-23-11897-s001.zip › Supplementary Figure S2.tif]
